# Chemical Control of Western Corn Rootworm (*Diabrotica virgifera virgifera* Le Conte, Coleoptera: Chrysomelidae) in Eastern Romania

**DOI:** 10.3390/insects16030293

**Published:** 2025-03-11

**Authors:** Roxana-Georgiana Amarghioalei, Nela Tălmaciu, Monica Herea, Ionela Mocanu, Paula-Lucelia Pintilie, Andreea-Sabina Pintilie, Elena Trotuș, Mihai Tălmaciu

**Affiliations:** 1 Agricultural Research and Development Station Secuieni–Neamt, Principala Street, No 371, 617415 Secuieni, Romania; roxana.amarghioalei@scda.ro (R.-G.A.); sabina.esanu@scda.ro (A.-S.P.); elena.trotus@scda.ro (E.T.); 2 Department of Plant Protection, Faculty of Horticulture, University of Life Sciences, Mihail Sadoveanu Alley, No 3, 700490 Iasi, Romania; mherea@uaiasi.ro (M.H.); imocanu@uaiasi.ro (I.M.); mtalmaciu@uaiasi.ro (M.T.)

**Keywords:** *Diabrotica*, attack, corn, chemical treatment

## Abstract

This paper aimed to determine the attack caused by WCR (*Diabrotica virgifera virgifera* Le Conte) and its control by chemical treatments on soil and vegetation. The research was carried out in eastern Romania between 2023 and 2024, where two trials were conducted in the experimental field of the Agricultural Research and Development Station Secuieni—Neamt, where three granular insecticides applied to the soil against larvae and three chemical insecticides used on vegetation against adults were tested. Among the granular insecticides tested, the Force G (tefluthrin 15 g/kg) insecticide stood out with the best results, significantly reducing the number of larvae/plant (1 larva/plant) and, at the same time, the absence of the swan neck symptom was also recorded. Regarding insecticides applied to vegetation, the best results were obtained with the insecticide Inazuma (acetamiprid 100 g/kg + lambda-cyhalothrin 30 g/kg), which recorded a very good efficacy in combating adults of the species (95.4%). In conclusion, applying chemical treatments to soil and vegetation is necessary to control the attack by western corn rootworm (WCR).

## Simple Summary

*Diabrotica virgifera virgifera* Le Conte is an important pest for the corn crop in Romania. The research carried out at the Agricultural Research and Development Station Secuieni (A.R.D.S.) consisted of determining the insect’s attack in the eastern part of the country and controlling it with the help of chemical treatments applied to the soil and vegetation. The results showed that the pest affected the plants in both the larval and adult stages. Granulated insecticides applied to the soil reduced the average number of larvae/plant and the frequency of attack they produced. Also, chemical insecticides applied to vegetation significantly reduced the number of adults/plant. Following the results, we conclude that the use of insecticides applied to soil and vegetation reduces the damage produced by *Diabrotica virgifera virgifera* Le Conte on corn.

## 1. Introduction

Corn (*Zea mays* L.) plays an important role in human nutrition and animal feed, and it is used as a raw material in various industries [1,2]. Due to its special phytotechnical and biological characteristics, corn occupies third place in terms of crop importance [2]. According to Faostat [3], in 2022, America ranked first in terms of area occupied by corn, with 75,883.8 thousand ha. Europe ranked fourth, with an area of 17,533.9 thousand ha. In 2022, Romania’s corn cultivation area was 2437 thousand ha, with an average production of 3297.7 kg/ha [3].

Various pathogens and pests affect crop yield, and their attacks reduce yield potential [2]. Western corn rootworm (*Diabrotica virgifera virgifera* Le Conte) (WCR) is one of the main pests of corn in Romania [4,5,6,7] and belongs to the order Coleoptera, family Chrysomelidae. It is native to North America, entered in Europe in 1992, and was reported in Romania in 1996 [8]. The spread of this pest is favored by the large areas cultivated with corn, especially by the practice of monoculture [9].

Studies on the biology of the species show that it has one generation per year, overwinters as an egg in the soil, and goes through all stages of development (egg, larva, pupa, and adult) [10,11].

The species attacks in both the larval and adult stages [12]. The larvae feed on the roots of corn plants, while the adults consume the plant’s aerial parts (leaves, panicle, silk, pollen, and grains in the milky phase) [13]. Plants affected by the larvae are recognized by the characteristic stem symptomatology, the “swan neck” [14]. The literature indicates that larvae cause significantly more damage to crops than adults [15]. Grain production can be reduced by up to 50% [16].

To prevent and combat this species, Grozea [17] mentions several methods, the most important of which is crop rotation. This is followed by applying chemical treatment to the seed and soil to control the larvae [18,19] and chemical treatment to vegetation to combat the adults [17]. Hill et al. [20] and Muma et al. [21] were the first to demonstrate the effect of soil insecticides against WCR.

Soil moisture (very dry or wet), pH, or soils with a high organic matter content contribute to varying the efficacy of soil-applied insecticides [22,23,24,25,26,27]. The ideal soil-applied insecticide should persist for 6–10 weeks, approximately the period between the insecticide application and the end of larval feeding. Soil-applied insecticides protect corn roots from feeding damage but do not affect WCR population density [23,28,29].

The chemical control of WCR focuses more on the larval stage, as it is more economically significant [30]. Granular insecticides applied to the soil and chemical seed treatments are widely used and have good efficacies in controlling WCR larvae [31,32], especially in monoculture corn fields. However, these methods only reduce larvae in the corn rows where insecticides have been applied, not those that develop outside the rows [28,33,34]. Insecticides applied to vegetation protect corn plants against WCR adults that feed excessively on corn silk [11]. This prevents grain filling and reduces the population of egg-laying adults, thus reducing the supply of larvae for the following year [35].

This work aimed to demonstrate the importance of chemical treatment in controlling larvae and adults of the species *D. virgifera virgifera* Le Conte by applying insecticides to soil and vegetation. The main objective was to establish the level of the attacks produced by this species, as well as the effectiveness of some chemical products with insecticidal action under the conditions of eastern Romania.

## 2. Materials and Methods

### 2.1. Experimental Site

The research was carried out in the Plant Protection Laboratory at the Agricultural Research and Development Station Secuieni—Neamt (A.R.D.S.), located at the geographical coordinates of 26°51′00″ east longitude and 46°51′15″ north latitude. Two large zones, or soil levels, were distinguished. In the upper part, corresponding to the forest area, there were soils from the cambisol and luvisol classes, and in the lower part (the forest–steppe area), there were soils from the chernisol class (chernozems; phaeozems with the subtypes typical, cambic, gleyic, alluvial, pelic). The boundary between the two areas was sinuous, due to the numerous interpenetrations, and difficult to specify in detail due to soils with transitional characteristics. The experimental fields of the A.R.D.S. Secuieni—Neamt are located on the first terrace of the Siret and in the Siret Meadow [36].

The climate is temperate continental, characterized by short springs, hot summers, and mild winters [37].

### 2.2. Field Experimental Design

The research was carried out from 2023 to 2024. Two corn trials were conducted with the Turda Star hybrid, a semi-early hybrid in the FAO 370 group created at the Agricultural Research and Development Station Turda. It is a trilinear hybrid resistant to low temperatures in the first part of the vegetation period, falling and breaking, drought, heat, grain cracking, and diseases and pests [38].

The first trial consisted of four treatments, one of which was a control, and involved testing granular insecticidal products applied to the soil at sowing to control WCR larvae. The control (V1) had no treatment, while in the other treatments, the following products were applied: V2—Force 1.5 G (tefluthrin 15 g/kg) at a dose of 15 kg/ha; V3—Picador 1.6 MG (cypermethrin 1.6%) at a dose of 12 kg/ha; and V4—Trika Expert (lambda-cyhalothrin 4 g/kg) at a dose of 15 kg/ha.

Force 1.5 G is a granular insecticide for soil application. The active substance is tefluthrin 15 g/kg. The action mode is by contact. It has a repellent effect, which provides long-lasting protection to plants in the early stages of development. The active substance (tefluthrin 15 g/kg) does not act systemically in plants and is not mobile in the soil. It is approved for corn crops to control *D. virgifera virgifera* Le Conte [39].

Picador 1.6 MG is a granular insecticide for soil application containing 1.6% cypermethrin, a non-mobile active substance belonging to the class of synthetic pyrethroids. Cypermethrin acts mainly by contact but also by ingestion. The insecticide is approved for the control of *D. virgifera virgifera* in corn and other species, such as *Agrotis segetum* and *Agriotes* spp. [40].

Trika Expert is a granular insecticide in the synthetic pyrethroid group. The active substance is lambda cyhalothrin 4 g/kg. It repels many pests through contact and ingestion. It is approved for corn for several pests, including *D. virgifera virgifera* Le Conte [41].

The second trial consisted of testing three insecticides applied to vegetation at the end of flowering to control WCR adults. In the control (V1), no treatment was applied, while in the other treatments, the following products were applied: V2—Decis Expert 100 EC (deltamethrin 100 g/L) at a dose of 0.075 L/ha; V3—Inazuma (acetamiprid 100 g/kg + lambda-cyhalothrin 30 g/kg) applied at a dose of 0.2 kg/ha; and V4—Fastac Active (alpha cypermethrin 50 g/L) at a dose of 0.6 L/ha.

Decis Expert 100 EC is a foliar insecticide that acts on harmful insects by contact and ingestion in their larval and adult stages. The active substance is deltamethrin (100 g/L). This synthetic pyrethroid paralyzes the insect’s nervous system. It has a rapid knockdown (shock) effect on insects and a repellent and anti-feeding effect, thereby protecting the treated plants. The product is approved for various crops and harmful insects [42].

Inazuma is a long-lasting systemic and contact insecticide. Its active substances are acetamiprid 100 g/kg and lambda-cyhalothrin 30 g/kg. The systemic active substance acetamiprid acts on pests by ingestion, and the contact active substance lambda-cyhalothrin also has a repellent effect. The insecticide is approved for corn for two of the most damaging insect pests: *Ostrinia nubilalis* Hbn. and *D. virgifera virgifera* Le Conte [43].

Fastac Active is a synthetic pyrethroid insecticide that controls adults, larvae, and insect eggs. The active ingredient, alpha-cypermethrin (50 g/L), has contact and ingestion action. In corn, it is approved only for the species *O. nubilalis*, but research has found that it also has good results in controlling the species *D. virgifera virgifera* [44].

The trials were arranged according to the randomized block method and were conducted in three replicates. The experimental treatment area was 22.4 square meters.

The preceding plant was corn. The soil work consisted of autumn plowing at a depth of 30 cm, and in the spring, two passes were made with a heavy disk harrow and a Terradisc. The preparation of the seedbed was carried out using the Atlas type combiner on the day before sowing. Fertilization was performed with a complex fertilizer of the N_20_P_20_K_0_ type at a dose of 200 kg/ha. Sowing was carried out in the first part of May 2023 and in the middle of April 2024. The distance between rows was 70 cm, and between plants in a row was 22 cm.

### 2.3. Establishing the Attack

Dynamic observations were made on each treatment to determine the larval attack on the root. Twenty-five plants were observed in three replicates for each treatment. The plants that showed attack, namely those in which the stem showed the swan neck symptom, were noted, and the attack frequency was calculated using the following formula:

F% = (n × 100)/N,

where n is the number of affected plants and N is the total number of analyzed plants [45].

Regarding the attack produced by adults, observations were made for each treatment, on leaves and silk, on 25 plants in three replicates, and the attack frequency was determined. The visual assessment of each treatment on 25 plants in three replicates determined the average number of adults/plant.

### 2.4. Determining the Efficacy of the Products Applied

The observations were carried out from late May to late June to establish the products’ efficacy in controlling the larvae. Five corn plants were removed from the soil, in three replicates, for each treatment, and the larvae identified on and around the roots were counted [46].

To determine the product efficacy applied to control adults, the average number of adults/plant was determined by the visual assessment of 25 plants in three replicates before chemical treatment on the vegetation and three days after its application.

Efficacy was calculated using the Abbott formula [47].

### 2.5. Statistical Analysis

All observations were carried out in three replicates for each treatment separately. The data obtained were calculated in Excel and statistically interpreted using the least significant differences test (LSD). The limit difference is the quantity that ensures the minimum value of surplus or deficit of production for transgression probabilities of 5%, 1%, and 0.1% [48]

## 3. Results

### 3.1. Climate Characterization of Years 2023–2024

The analyzed climatic data come from the unit’s meteorological station, Wireless Vantage Pro 2 Plus (SC Rom Tech SRL, Sibiu, Romania), located at the Agricultural Research and Development Station Secuieni near the experimental fields. The data recorded by the meteorological station are the maximum daily temperature (°C), the minimum daily temperature (°C), the average daily temperature (°C), the daily precipitation (mm), the relative humidity (%), the maximum daily soil temperature (°C), the minimum daily soil temperature (°C), and the average daily soil temperature (°C). To characterize the years from a climatic point of view, we used the data relating to the average air temperature (°C) and precipitation (mm) [49].

**Figure 1:**
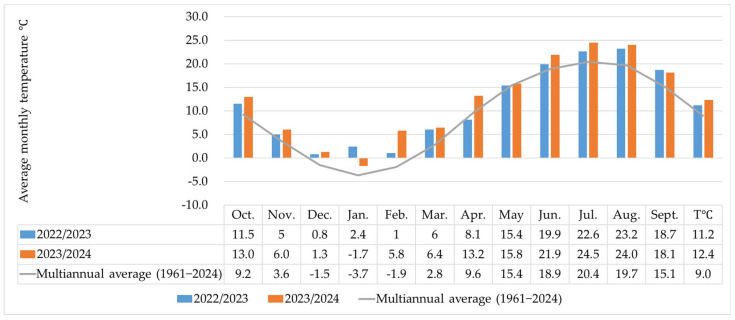
Average monthly temperatures and multiannual averages recorded in 2022/2023 and 2023/2024.

The temperatures recorded over the two years demonstrate a clear warming trend in the climate. The deviation from the multiannual average (9.0 °C) was 2.3 °C in 2023 and 3.4 °C in 2024. The monthly deviations in 2022/2023 were between —1.5 °C (April) and +6.1 °C (January), and in 2023/2024, the monthly deviations were between 0.4 °C (May) and 7.7 °C (February) (Figure 1).

**Figure 2:**
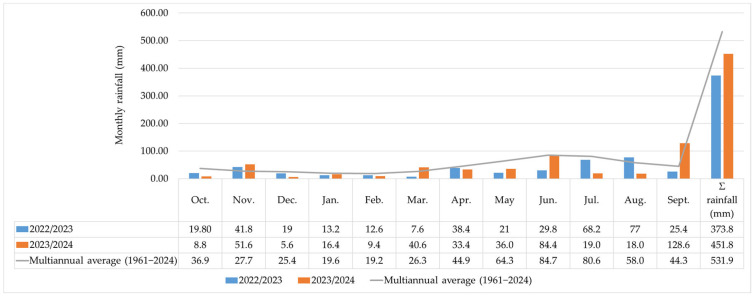
Monthly rainfall and multiannual averages recorded in 2022/2023 and 2023/2024.

Regarding rainfall, 2022/2023 and 2023/2024 were dry. The deviation from the annual amount of rainfall (531.9 mm) was —158.1 mm in 2023 and —80.1 mm in 2024. The monthly deviations ranged from —54.9 mm (June) to 19.0 mm (August) in 2022/2023 and from —61.6 mm (July) to 84.3 mm (September) in 2023/2024 (Figure 2).

### 3.2. Influence on WCR Larvae Density After Application of Granular Insecticides to Soil

**Figure 3:**
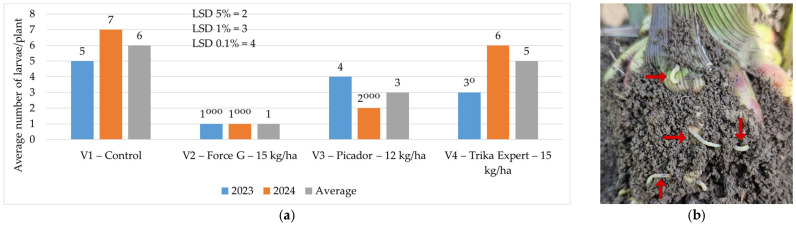
(**a**) Average number of larvae/plant 2023—2024; (**b**) larvae/plant at A.R.D.S. Secuieni—Neamt (original; red arrows indicates the larvae) (*p* < 0.001—^ooo^ negative very significant; *p* < 0.05—^o^ negative significant, LSD).

Where granular insecticides were applied to the soil with sowing, the average number of larvae/plant was between 1 larva/plant and 4 larvae/plant in 2023 and between 1 larva/plant and 6 larvae/plant in 2024, compared to the control, where average number of larvae/plant was 5 larvae/plant (2023) and 7 larvae/plant (2024). During the two years of experimentation, the average number of larvae ranged from one larva/plant to five larvae/plant. The best results were obtained where the granular insecticide Force G (15 kg/ha) was applied to the soil, ensuring the good protection of the corn roots against the WCR larvae (Figure 3).

**Figure 4:**
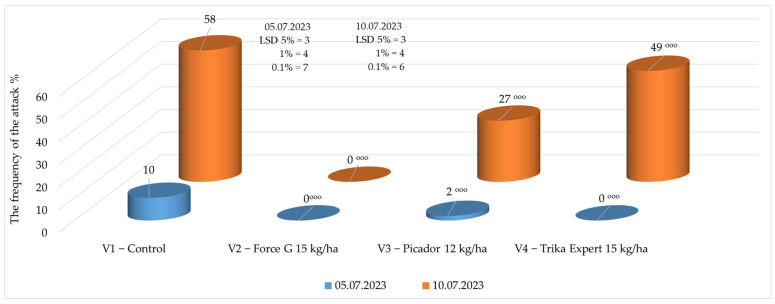
Frequency of swan neck symptom in 2023. (*p* < 0.001—^ooo^ negative very significant, LSD).

In 2023, upon the first determination of the frequency of the swan neck symptom, it was found to be reduced, ranging between 0% and 2% in the soil-treated variants and 10% in the control without soil treatment (Figure 4).

In the second determination, after the recording of a summer storm, the swan neck symptom was much more visible in the crop, ranging between 0% and 49% where the granular insecticides were applied to the soil, compared to 58% recorded in the control. The best results were obtained for the treatment where the granular insecticide Force G (15 kg/ha) was applied to the soil, with the insecticide ensuring the good protection of the roots compared to the other products applied [50] (Figure 4).

**Figure 5:**
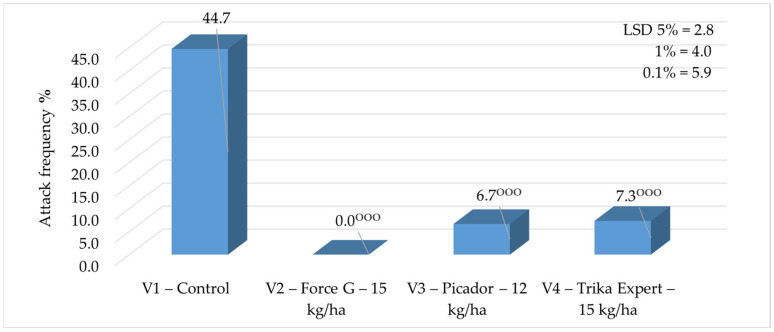
Frequency of swan neck symptom in 2024 (*p* < 0.001—^ooo^ negative very significant, LSD).

In 2024, the frequency of the swan neck symptom recorded values ranged from 0%, as recorded where the granular insecticide Force G (15 kg/ha) was applied to the soil, and 44.7%, as in the control (Figure 5).

**Figure 6:**
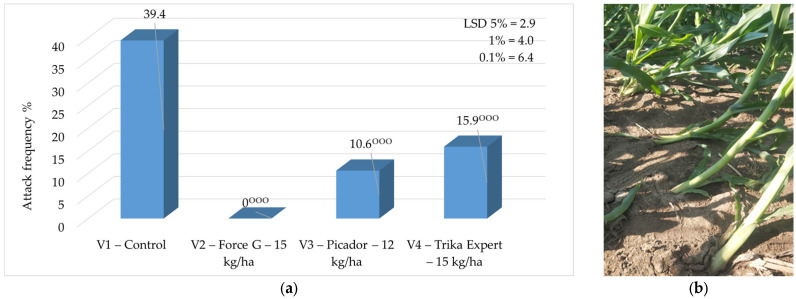
(**a**) Average frequency of swan neck symptom, 2023—2024; (**b**) image of swan neck symptom at A.R.D.S Secuieni—Neamt (original) (*p* < 0.001—^ooo^ negative very significant, LSD).

On average, during the two years of experimentation, the best results were obtained where the granular insecticide Force G (15 kg/ha) was applied to the soil. The frequency of the swan neck symptom ranged between 0% (Force G) and 39.4% (control) (Figure 6).

### 3.3. Influence on WCR Adult Density After Application of Chemical Treatment to Vegetation

In 2023, the frequency of adult attack on leaves was 50%, and 100% on silk. In 2024, the attack on leaves was 59%, and on silk, 89%.

**Figure 7:**
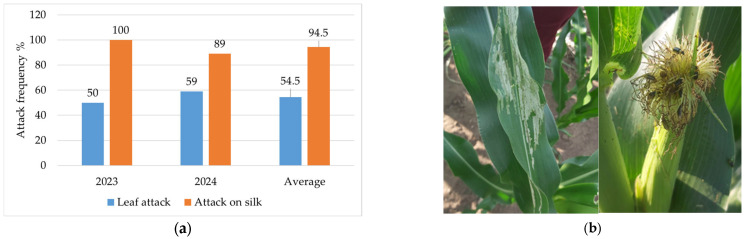
(**a**) Frequency of attack by WCR adults on leaves and silk, 2023—2024; (**b**) image of attack on leaves and silk (original).

On average, over the two years of monitoring, WCR adults attacked the leaves at a rate of 54.5%, and the frequency of attack on silk was 94.5%, wholly or partially consuming the corn silk (Figure 7).

**Figure 8:**
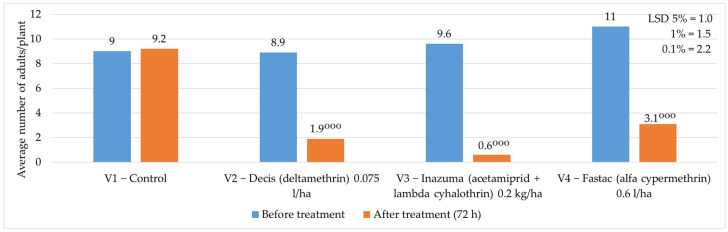
The average number of adults/plant before and after the application of chemical treatment to vegetation, 2023. (*p* < 0.001—^ooo^ negative very significant, LSD).

Regarding the average number of adults/plant, in 2023, before applying the chemical vegetation treatment, it was between 8.9 adults/plant and 11 adults/plant. Determinations made three days after the application of the chemical treatment showed that the average number of adults/plant was reduced, being between 0.6 adults/plant and 3.1 adults/plant where the treatment was applied, while in the control, the average number of adults/plant was 9.2 (Figure 8).

**Figure 9:**
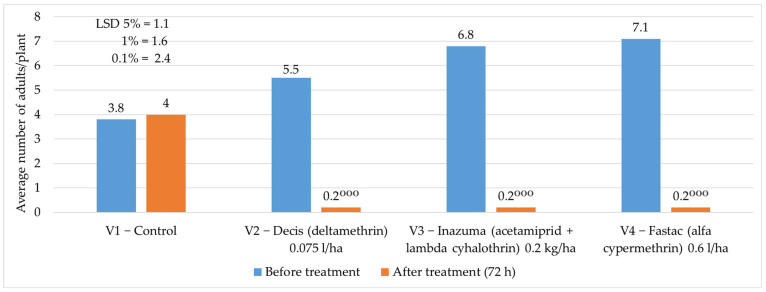
The average number of adults/plant before and after the application of chemical treatment to vegetation, 2024. (*p* < 0.001—^ooo^ negative very significant, LSD).

In 2024, the average number of adults/plant before the chemical vegetation treatment recorded values ranging between 3.8 adults/plant and 7.1 adults/plant. After the treatment, it was found that the applied insecticides reduced the number of adults to 0.2 adults/plant compared with 4 adults/plant registered at the control (Figure 9).

**Figure 10:**
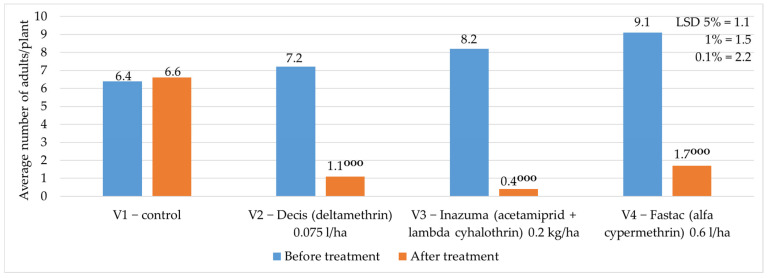
The averages of the years 2023—2024 regarding the number of adults/plant before and after the application of chemical treatment to vegetation. (*p* < 0.001—^ooo^ negative very significant, LSD).

During 2023–2024, the average number of adults/plant was between 6.4 adults/plant and 9.1 adults/plant before applying the chemical treatment to vegetation, and after the treatment, the average number of adults/plant ranged between 0.4 adults/plant and 1.7 adults/plant in the treated variants, and 6.6 adults/plant were registered in the control variant (Figure 10).

**Figure 11:**
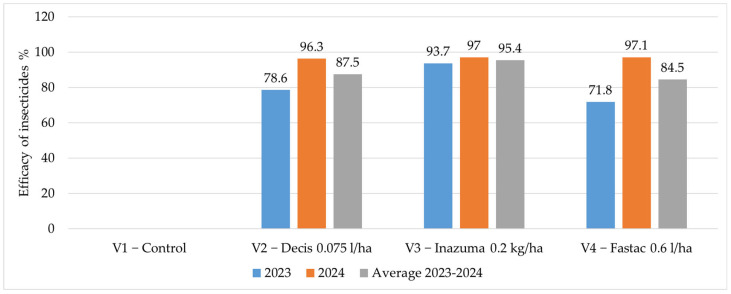
The efficacy of the insecticides applied to control the WCR adults (E %).

Regarding the efficacy of the insecticides applied to vegetation to control the WCR adults, the best results were obtained in the treatment where Inazuma (0.2 kg/ha) was used, with an average efficacy of 95.4% (Figure 11).

## 4. Discussion

During the two years of monitoring at the Agricultural Research and Development Station Secuieni (A.R.D.S.), the western corn rootworm (WCR) attacked in the larval and adult stages. The larval attack on the roots manifested as the presence of the swan neck symptom, and the adult attack manifested as the consumption of leaves and silk, as well as grains at the top of the cobs.

Various chemical treatment options are available in Europe, including seed or soil treatments against larvae before or during sowing. Foliar insecticides can be applied to reduce adult populations so that the crop is not compromised, or to reduce oviposition, but their efficacy depends on the timing of application, for example, at the beginning of the season during the pre-oviposition period [51]. Foliar insecticides prevent or control the WCR adult attack on silk during pollination. They also control the damages to leaves, which can be wholly or partially torn off in a massive attack [52].

Our research demonstrates that the chemical treatment of soil and vegetation is essential to control WCR larvae and adults, the number of larvae/plant being 1 larva/plant in the treatments, compared to the control where 6 larvae/plant were recorded, and the number of adults being reduced after treatment to 0.4 adults/plant, compared to the control where 6.6 adults/plant were recorded. This is also supported by Ciobanu et al. [53], who recommend the application of granular insecticides to the soil, especially in monoculture (Force 1.5 G). Chemical seed treatment with insecticides also reduces the number of plants attacked. Chemical treatment against adults should be applied to reduce the adult ovipositing population, determining the larvae population next year [53].

Rozen and Ester [54] analyzed the control strategies used in the USA and EU against WCR. They concluded that rotation is the main method of prevention and control of the WCR attack, the practice of corn monoculture being the primary source of pest multiplication. Another method to prevent and control the attack is seed and soil treatment with insecticides [54].

The results obtained at A.R.D.S. Secuieni—Neamt show that granular insecticides applied to the soil significantly reduced the number of larvae as well as the attack produced by them, and that the best results were obtained where the granular insecticide Force 1.5 G was applied. This is also supported by Florian [55], who tested the same insecticide (Force 1.5 G) and found that it reduced the frequency of attack by WCR larvae, as in our study. The same insecticide was also tested by Tălmaciu et al. [56], and their results demonstrated that this product reduces the population of WCR larvae and adults. Also, Voros [31] states that the best results were obtained where the granular insecticide Force 1.5 G (tefluthrin) was applied to the soil at the same time as sowing, with the lowest number of larvae and the least attack on the roots.

Research similar to that at A.R.D.S. Secuieni—Neamt was also carried out in Italy, where the effect of soil-applied insecticides to control the WCR larval attack was monitored. It was observed that the application of insecticides significantly reduced the density of larvae/plant, which contributed to a decrease in root attack and a reduction in the frequency of plants with the swan neck symptom [57].

In 2016–2017, area-wide pest management strategies were evaluated in northeastern Italy. One of the approaches compared corn grown in monoculture for several years, where seed treatment was used, and on vegetation and corn grown in rotation. It was found that the species density was higher in corn with chemical treatments than in the rotation corn, demonstrating that crop rotation is the most effective method to control the pest [58].

Ferracini et al. [59] evaluated the effect of different chemical control strategies to reduce the damage caused by larvae in corn crops. Different insecticides from the pyrethroid, neonicotinoid, or organophosphorus classes were compared with an untreated variant. The results showed that both the seed and soil application of insecticides contributed to a significant reduction in WCR larvae density. In particular, clothianidin applied to the seed (systemic) and tefluthrin applied at sowing resulted in an 18% and 19% yield increase, respectively, compared to the control.

Ciobanu et al. [60] conducted research on the control of WCR adults by applying insecticides to vegetation and concluded that they had high efficacy. Similar research was also conducted in our study, demonstrating the importance of using chemical treatment on vegetation, which provides good protection against WCR adults.

Studies have shown that the long-term use of chemical insecticides has led to the development of resistance in populations of *D. virgifera virgifera*, affecting the efficacy of treatments and requiring more complex management strategies [35]. To prevent this, an integrated approach to control methods is needed, including crop rotation, the cultivation of tolerant hybrids [35], or biological control using nematodes [61].

## 5. Conclusions

In conclusion, our study showed that the WCR species attacked corn roots, leaves, and silk in both stages of development (larva and adult). Also, this study demonstrated the importance of applying chemical treatments to the soil and vegetation, which achieved a good result in the control of WCR larvae and adults.

## Data Availability

The data presented in this study are available on request from the corresponding authors.
